# HDAC4 inhibition disrupts TET2 function in high-risk MDS and AML

**DOI:** 10.18632/aging.103605

**Published:** 2020-07-29

**Authors:** Feiteng Huang, Jie Sun, Wei Chen, Xin He, Yinghui Zhu, Haojie Dong, Hanying Wang, Zheng Li, Lei Zhang, Samer Khaled, Guido Marcucci, Jinwen Huang, Ling Li

**Affiliations:** 1 Department of Hematology, Sir Run Run Shaw Hospital, School of Medicine, Zhejiang University, Hangzhou 310016, China; 2 Department of Hematological Malignancies Translational Science, Gehr Family Center for Leukemia Research, Hematologic Malignancies and Stem Cell Transplantation Institute, Beckman Research Institute, City of Hope Medical Center, Duarte, CA 91010, USA; 3 The Integrative Genomics Core, Beckman Research Institute, City of Hope Medical Center, Duarte, CA 91010, USA; 4 Department of Hematology and Hematopoietic Cell Transplantation (HCT), Beckman Research Institute, City of Hope Medical Center, Duarte, CA 91010, USA

**Keywords:** myelodysplastic syndromes, acute myeloid leukemia, histone deacetylase, TET2

## Abstract

Aberrant DNA methylation often silences transcription of tumor-suppressor genes and is considered a hallmark of myeloid neoplasms. Similarly, histone deacetylation represses transcription of genes responsible for cell differentiation/death. A previous clinical study suggested potential pharmacodynamic antagonism between histone deacetylase inhibitors (HDACi) and DNA hypomethylating agents (HMA). Herein, to determine such antagonism, we used MDS/AML lines and NHD13 transgenic mice, and demonstrated that treatment with the pan-HDACi suberoylanilide hydroxamic acid (SAHA) significantly decreased TET2 expression and global 5-hydroxymethylcytosine (5hmC) levels. Mechanistically, our RNAi screen revealed that HDAC4 was responsible for maintaining TET2 levels. Accordingly, HDAC4 knockout reduced expression levels of MTSS1, a known TET2 target, an event associated with decreased 5hmC enrichment on the MTSS1 enhancer. Retrospective analysis of GEO datasets demonstrated that lower HDAC4 levels predict worse prognosis for AML patients. In an MDS-L xenografted immunodeficient mouse model, vitamin C co-treatment prevented TET2 loss of activity seen following SAHA treatment. Accordingly, vitamin C co-treatment further reduced MDS-L cell engraftment relative to SAHA alone. In summary, our findings suggest that co-administration of a TET2 agonist with pan-HDACi treatment could effectively counter potential diminution in TET2 activity resulting from pan-HDACi treatment alone, providing a rationale for evaluating such combinations against high-risk MDS/AML.

## INTRODUCTION

DNA methylation is an essential epigenetic modification in mammalian cells. Aberrant hypermethylation of promoter CpG islands can repress gene transcription and promote tumorigenesis [1, 2]. DNA hypomethylating agents (HMA) block activity of DNA methyltransferases and reportedly reverse aberrant DNA methylation in some cancers [3]. Besides passive DNA demethylation seen following HMA treatment, DNA demethylation can also occur actively through Tet methylcytosine dioxygenase 2 (TET2), which oxidizes methylated cytosine (5mC) to 5-hydroxymethylcytosine (5hmC) [4]. Remarkably, TET2 is frequently mutated in hematological malignancies [5]. TET2 loss-of-function leads to DNA hypermethylation and dysregulates gene expression in hematopoietic stem/progenitor cells (HSPCs), resulting in aberrant hematopoiesis [6]. Thus, TET2 safeguards against HSPC transformation. However, a major subset of myelodysplastic syndromes (MDS) cases harboring wild type (WT) TET2 show significantly lower global 5hmC levels than do healthy donors [7], suggesting that other factors regulate TET2 function, such as protein acetylation, as reported in our previous study [8].

MDS are clonal hematopoietic disorders characterized by morphological dysplasia, cytopenias and increased risk of transformation to acute myeloid leukemia (AML) [9]. MDS and AML are most prevalent in the elderly, with the median age at diagnosis between 65 and 75 years [9]. MDS is a highly heterogeneous disease, and patients are categorized into five risk groups based on the Revised International Prognostic Scoring System (IPSS-R) [10]. Over the past decades, mounting evidence indicates that aberrant epigenetic regulation is critical for pathogenesis of high-risk MDS and AML [11, 12]. Histone deacetylases (HDACs), as epigenetic erasers, catalyze removal of histone acetyl groups, thereby regulating gene expression [13]. Many clinical trials are ongoing to evaluate the efficacy of HDAC inhibitors (HDACi) in hematologic malignancies [14]. Moreover, to improve clinical outcomes, HDACi are commonly used in combination with other treatment, such as HMA [15]. Notably, a phase 2 randomized trial combining HMA with HDACi against MDS/AML indicated reduced global DNA demethylation relative to HMA monotherapy, suggesting antagonism between HMA and HDACi [16]. Thus, understanding HDACi function in DNA methylation is required to design more effective HDACi associated combination therapy.

Here, we show that administration of suberoylanilide hydroxamic acid (SAHA), an FDA-approved pan-HDACi, significantly reduced TET2 activity in MDS/AML cells. RNAi screening revealed that HDAC4 expression maintains TET2 activity and accordingly, HDAC4 inhibition disrupted active DNA demethylation. Finally, treatment with vitamin C in combination with SAHA not only prevented TET2 loss of activity but also significantly decreased MDS-L cell growth in immunodeficient mice. Overall, our work indicates that restoring TET2 function may overcome the negative effects of HDACi administration on DNA demethylation and also provides a rationale to further test combined treatment with a TET2 agonist plus an HDACi against high-risk MDS or AML.

## RESULTS

### HDACi treatment decreases total 5hmC levels and TET2 expression in a murine MDS model

**Figure 1:**
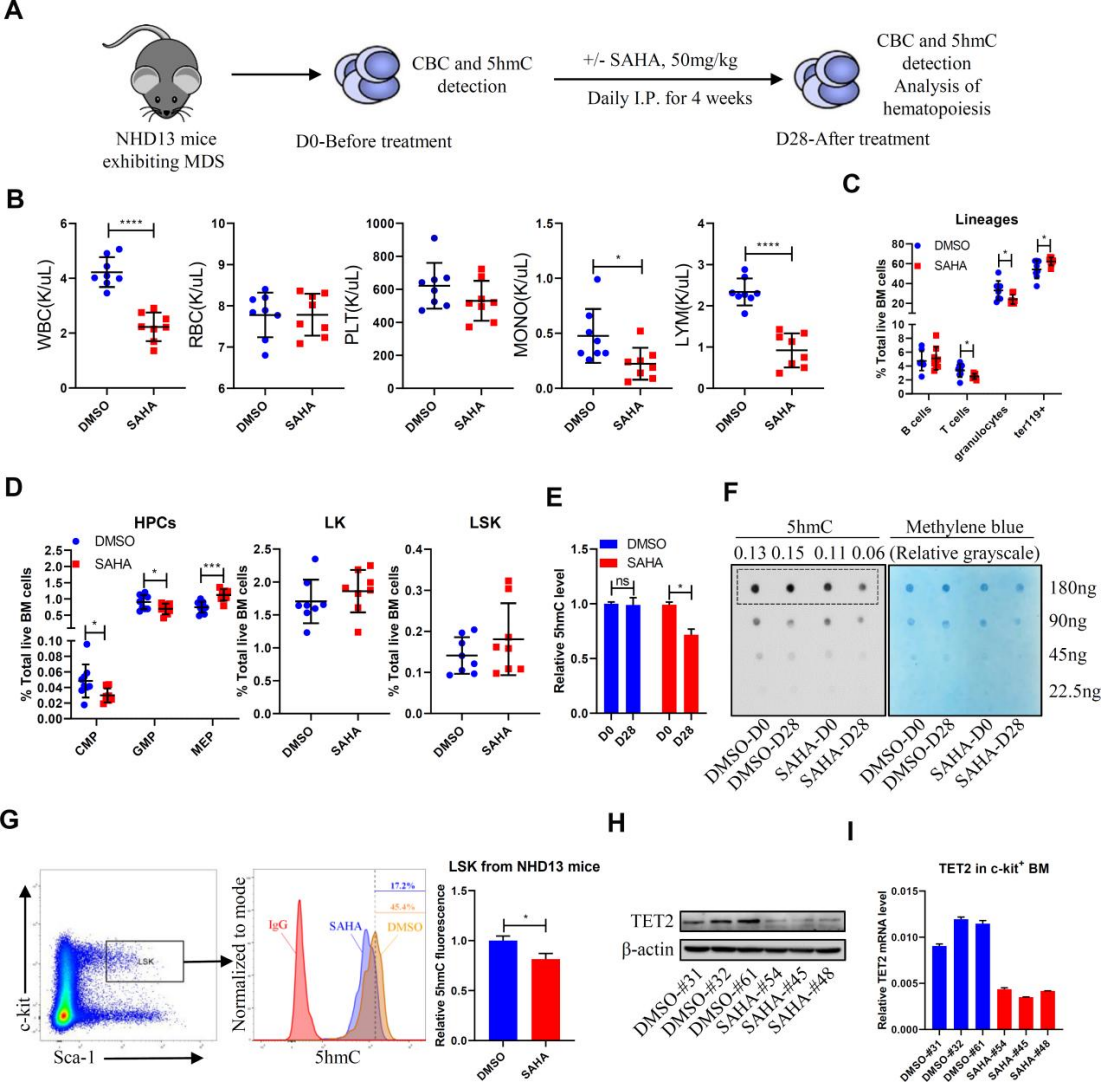
**SAHA treatment reduces TET2 and 5hmC levels in HSPCs of NHD13 mice.** (**A**) NHD13 mice were divided into two groups of matched gender and age. Peripheral blood (PB) was sampled before treatment, and mice were then treated 4 weeks with SAHA (50mg/kg/day, I.P., n = 8) or vehicle (5% DMSO in 20% cyclodextrin, I.P., n = 8). After treatment, mice were euthanized, and PB and BM were collected. (**B**) CBC as indicated in NHD13 mice analyzed after 4 weeks of treatment. (**C**) Hematopoietic lineage subset frequencies in BM of NHD13 mice after 4 weeks of indicated treatment. (**D**) Hematopoietic progenitor subset frequencies in BM of NHD13 mice after 4 weeks of indicated treatment. (**E**, **F**) 5hmC levels in MNCs of PB from NHD13 mice before or after treatment, as determined by ELISA (**E**) and dot blot (**F**). Methylene blue staining served as a loading control. 5hmC levels were normalized against loading of total DNA. (**G**) 5hmC levels in LSK cells of BM from treated NHD13 mice (n = 3), as determined by intracellular staining with a 5hmC-specific antibody. (**H**) Western blot showing TET2 protein levels in c-kit+ cells from BM of NHD13 mice treated as indicated. (**I**) RT-qPCR detection of TET2 mRNA levels in c-kit+ cells from BM of NHD13 mice treated as indicated.

We first chose to assess effects of in-vivo treatment with the pan-HDACi SAHA in NUP98-HOXD13 (NHD13) transgenic mice, which display severe symptoms resembling human MDS and die of cytopenias or transformed leukemia in 8-12 months [17]. After confirming MDS development, as evidenced by leukopenia and anemia, we treated NHD13 mice with SAHA (I.P. 50mg/kg) or vehicle control (Figure 1A) for 4 weeks. SAHA treatment did not reverse cytopenias but decreased the number of granulocytes and lymphocytes in bone marrow (BM) and peripheral blood (PB) (Figure 1B, 1C). SAHA treatment did not alter LK (Lin^-^Sca1^-^c-kit^+^) or LSK (Lin^-^Sca1^+^c-kit^+^) frequency, but decreased frequencies of common myeloid progenitors (CMPs) and granulocyte-monocyte progenitors (GMPs) (Figure 1D). We observed a modest increase in the number of megakaryocyte erythrocyte progenitors (MEPs) and ter119^+^ erythroid cells in BM of the SAHA-treated mice, but anemia was not reversed (Figure 1C, 1D).

We next asked whether SAHA treatment altered DNA demethylation in NHD13 mice. We found that after 4 weeks of SAHA treatment, mice showed significantly decreased 5hmC levels in PB mononuclear cells (MNCs) relative to vehicle-treated controls (Figure 1E, 1F). Moreover, intracellular staining confirmed reduced 5hmC levels in LSKs of SAHA- versus vehicle-treated NHD13 mice (Figure 1G). We further assessed TET2 protein and mRNA levels after SAHA treatment and found that both were lower in c-kit^+^ BM cells from SAHA- relative to vehicle-treated mice (Figure 1H, 1I).

### HDACi treatment reduces total 5hmC and TET2 expression levels in MDS/AML lines

**Figure 2:**
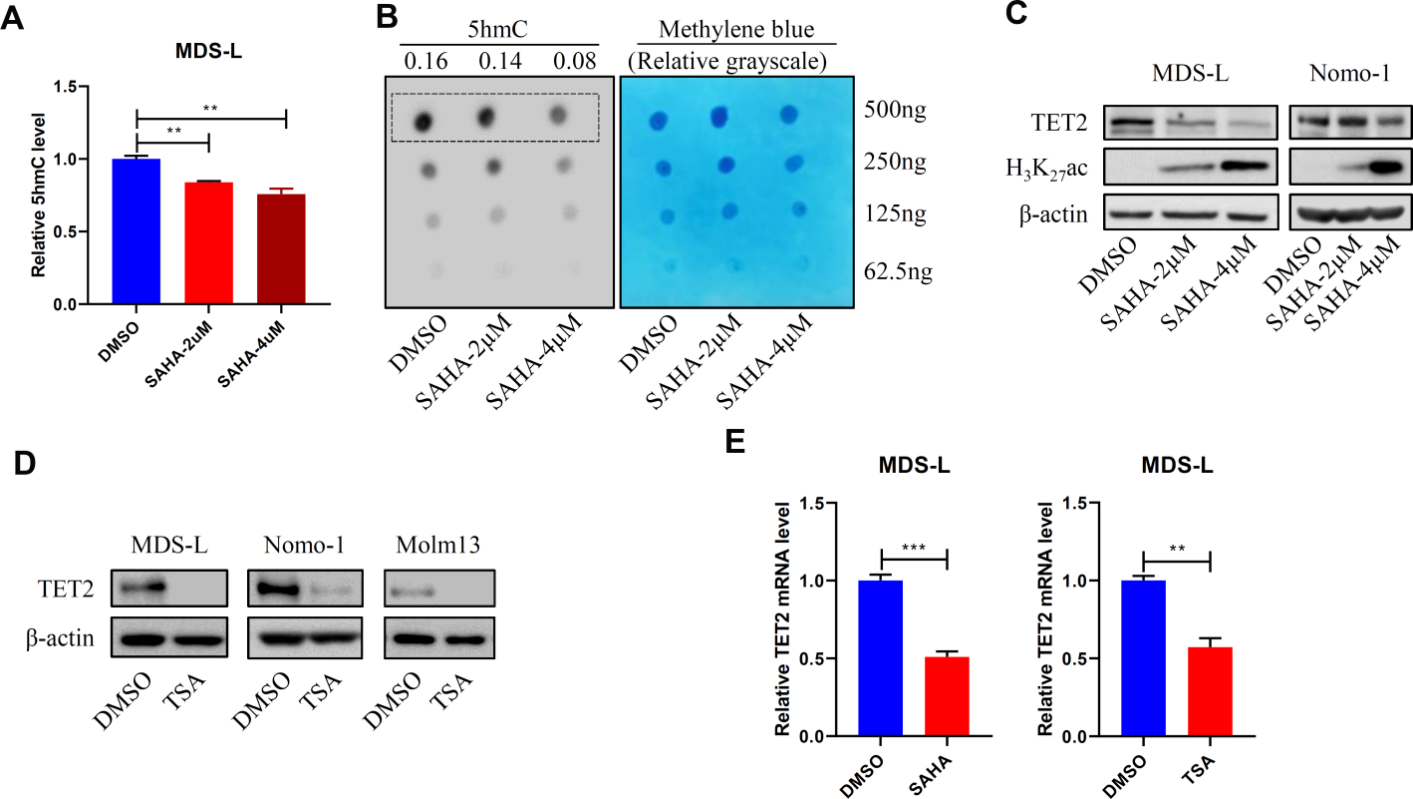
**HDACi treatment promotes DNA hypermethylation.** (**A**, **B**) MDS-L cells were treated with 2 or 4 μM SAHA for 24 hours, and then 5hmC levels were determined by ELISA (**A**) or dot blot (**B**). (**C**, **D**) MDS-L, Nomo-1 or Molm13 cells were treated with 2 or 4 μM SAHA (**C**) or 1 μM TSA (**D**) for 24 hours, and then TET2 protein levels were determined by Western blot. In (**C**) H3K27Ac served as a positive control indicative of HDAC inhibition. (**E**) TET2 mRNA levels in 2 μM SAHA- or 1 μM TSA-treated MDS-L cells, as detected by RT-qPCR.

We next treated human MDS and AML cell lines ex-vivo with SAHA. Treatment significantly decreased total 5hmC levels in MDS-L cells (Figure 2A, 2B), a line derived from an MDS patient [18], as well as TET2 expression levels in MDS-L cells and in human AML cell lines (Figure 2C and Supplementary Figure 1A). As a positive control, SAHA treatment also increased levels of histone H3K27 acetylation (H_3_K_27_Ac) (Figure 2C, Supplementary Figure 1A). Similarly, treatment of MDS-L cells with a different pan-HDACi, Trichostatin A (TSA), reduced TET2 protein levels (Figure 2D). SAHA or TSA treatment also reduced TET2 mRNA levels (Figure 2E and Supplementary Figure 1B), indicating that HDACi treatment downregulates TET2 at the transcriptional level. Expectedly, SAHA or TSA treatment ex-vivo reduced MDS-L and Nomo-1 cell viability (Supplementary Figure 2A, 2B). Overall, these results indicate that although HDACi treatment has an anti-cancer effect, it can also significantly downregulate TET2 activity, impairing DNA demethylation capacity and potentially counteracting a therapeutic effect.

### HDAC4 activity is required to maintain TET2 levels

**Figure 3:**
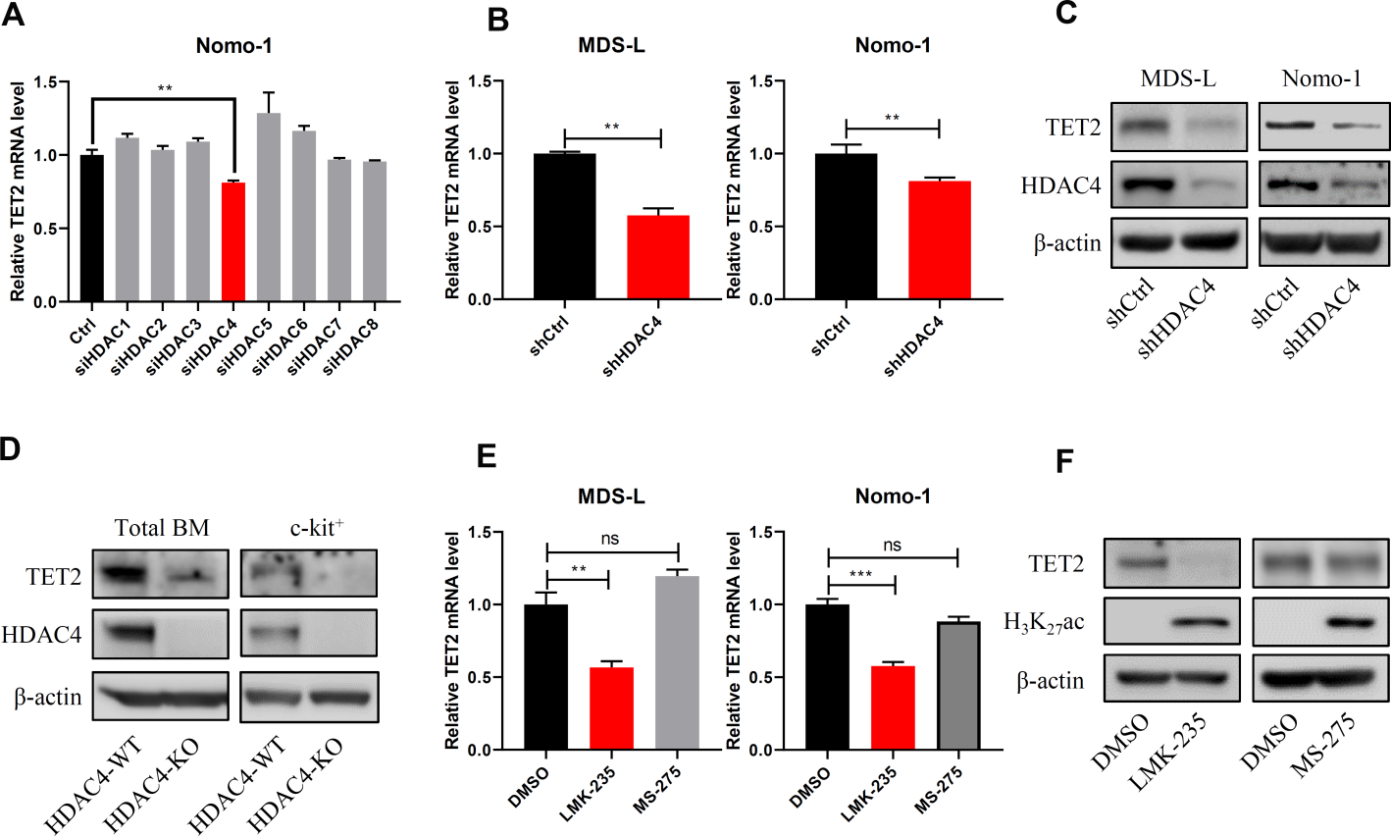
**HDAC4 knockdown decreases TET2 expression.** (**A**) Nomo-1 cells were transduced with siRNAs targeting indicated HDAC genes, and TET2 expression was determined by RT-qPCR. (**B**, **C**) HDAC4 knockdown using lentivirus to deliver shRNA to MDS-L and Nomo-1 cells. TET2 expression was determined by RT-qPCR (**B**) or Western blot (**C**). (**D**) Total BM and c-kit^+^ cells were harvested from HDAC4 WT or KO mice, and TET2 protein levels were determined by Western blot. (**E**) MDS-L and Nomo-1 cells were treated with 5 μM LMK-235 or 1 μM MS-275 for 24 hours, and TET2 expression was determined by RT-qPCR. (**F**) MDS-L cells treated with LMK-235 or MS-275 were analyzed by Western blotting for TET2 expression. H3K27Ac served as a positive control.

We next asked which HDAC is responsible for maintaining TET2 activity. Utilizing siRNAs targeting specific genes that encode HDACs, we found that only knockdown of HDAC4, a class II HDAC [13], decreased TET2 mRNA levels (Figure 3A). To validate results of the RNAi screen, we then knocked down HDAC4 in MDS-L or Nomo-1 cells by lentiviral delivery of shRNA and in both observed decreased TET2 mRNA and protein levels (Figure 3B, 3C). Next, we measured TET2 expression levels in BM of HDAC4 knockout (KO) mice and found relatively decreased TET2 protein levels (Figure 3D). To confirm that HDAC4 regulates TET2 expression, we treated MDS-L cells with the HDAC4/5 inhibitor LMK-235 and observed marked downregulation of TET2 mRNA and protein levels relative to vehicle controls. In contrast, treatment of MDS-L cells with the HDAC1/HDAC3 inhibitor MS-275 did not alter TET2 levels, although H_3_K_27_Ac levels were significantly increased relative to untreated cells (Figure 3E, 3F).

**Figure 4:**
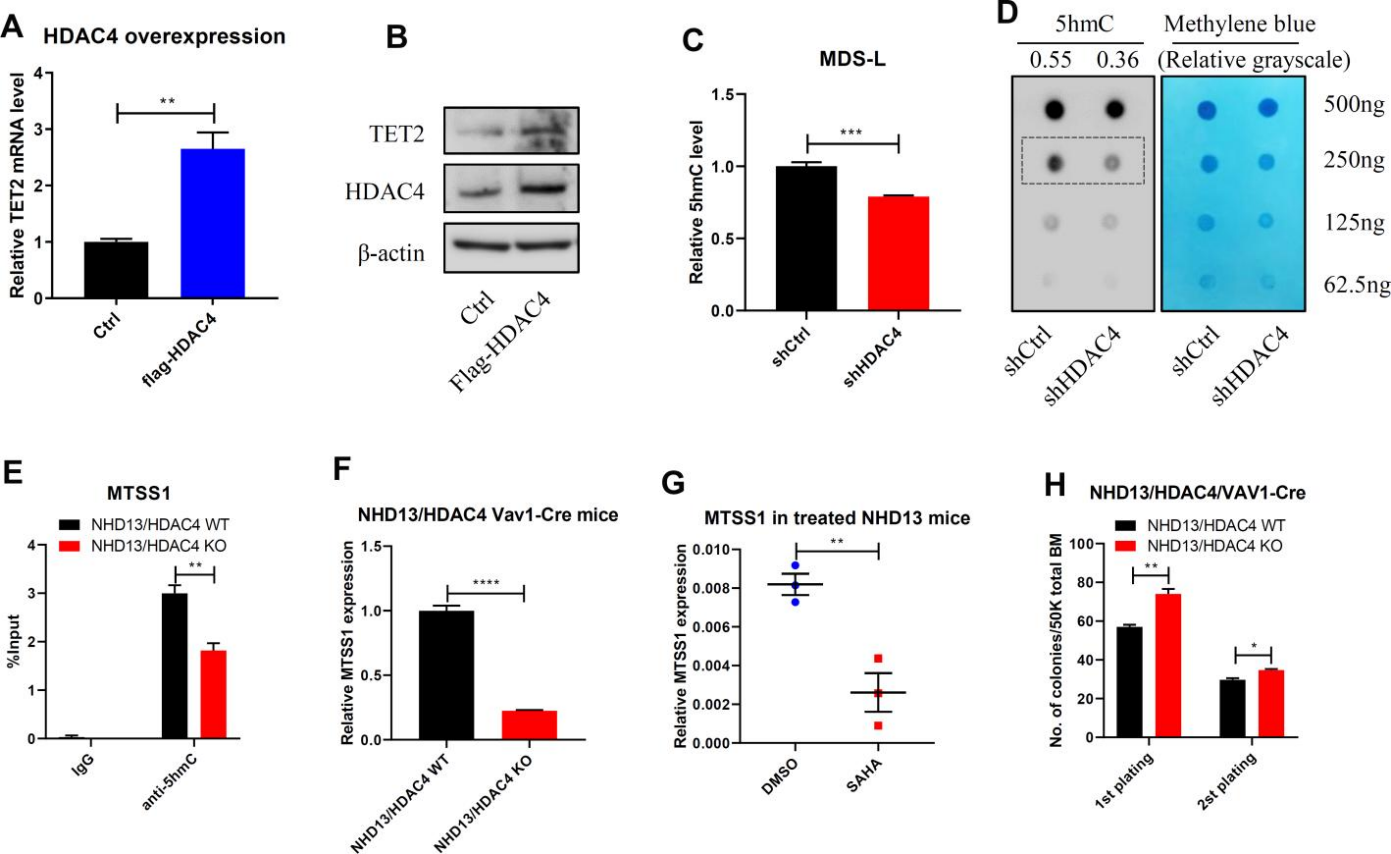
**HDAC4 is required to maintain TET2 activity.** (**A**, **B**) HDAC4 was overexpressed in 293T cells and TET2 expression determined by RT-qPCR (**A**) or Western blot (**B**). (**C**, **D**) After HDAC4 knockdown using lentivirus to deliver shRNA to MDS-L cells, 5hmC levels were determined by ELISA (**C**) or dot blot (**D**). (**E**) hMeDIP-qPCR analysis of specific 5hmC enrichment on Chr15: 58979469-58979554, enhancer region of MTSS1 in c-kit^+^ cells from BM of NHD13/HDAC4 WT or NHD13/HDAC4 KO mice. Bars represent mean enrichment over input. (**F**) MTSS1 mRNA levels in c-kit^+^ cells from BM of NHD13/HDAC4 WT or NHD13/HDAC4 KO mice. (**G**) RT-qPCR detection of MTSS1 mRNA levels in c-kit^+^ cells from BM of NHD13 mice treated with SAHA or vehicle control. (**H**) Serial replating of NHD13/HDAC4 WT or NHD13/HDAC4 KO cells. Total BM cells from each these mice were seeded in methylcellulose medium, and colonies were counted on day 7.

Notably, overexpression of HDAC4 upregulated TET2 expression levels (Figure 4A, 4B). We also observed decreased total 5hmC levels in HDAC4 knockdown MDS-L cells relative to controls (Figure 4C, 4D). MTSS1 is a reported TET2 target and tumor suppressor gene [19]. Thus, we determined MTSS1 expression levels and evaluated potential changes in 5hmC enrichment on the MTSS1 enhancer region in HDAC4 KO cells. As expected, HDAC4 KO mice showed relatively decreased 5hmC enrichment on the MTSS1 enhancer (Chr15: 58979469-58979554) (Figure 4E), a change associated with reduced MTSS1 expression (Figure 4F). Moreover, MTSS1 expression levels decreased in NHD13 mice following SAHA treatment (Figure 4G). Phenotypically, NHD13/HDAC4 KO BM cells showed significantly enhanced colony formation capacity relative to NHD13/HDAC4 WT BM cells (Figure 4H). Overall, these results indicate that HDAC4 maintains TET2 activity and that HDAC4 deficiency may promote MDS cell growth.

### HDAC4 expression levels correlate with TET2 levels in high-risk MDS/AML

**Figure 5:**
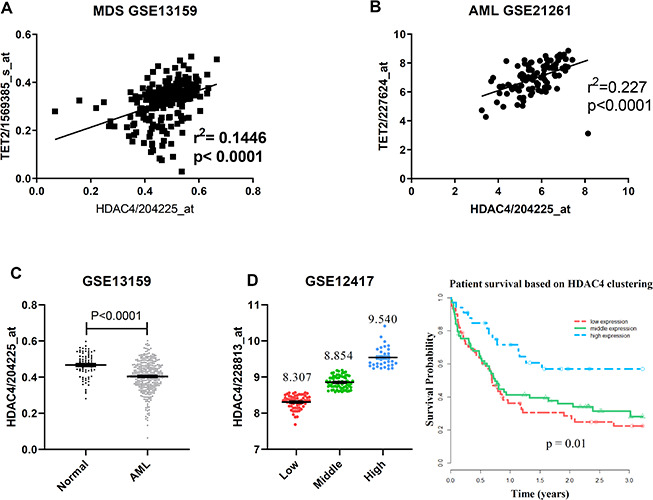
**TET2 and HDAC4 expression is positively correlated in high-risk MDS/AML.** (**A**, **B**) Microarray expression data from total BM of MDS (**A**, n = 206) or mononuclear AML (**B**, n = 96) cells. P value was calculated by Pearson r correlation. (**C**) HDAC4 expression data from total BM from healthy donors (n = 73) or normal karyotype AML patients (n = 351). P value was calculated by unpaired t test. (**D**) Overall survival in 163 normal karyotype AML patients based on clustering of HDAC4 expression. Patients were separated into 3 expression groups—low (n = 61), middle (n = 69) and high (n = 33)—based on a standard K-means clustering approach. Kaplan-Meier curves were generated and log-rank p-values calculated to assess statistical significance.

We next analyzed public accessible GEO dataset and observed a positive correlation between HDAC4 and TET2 levels within a MDS cohort (GSE13159 [20, 21]) (Figure 5A). This correlation was confirmed in another AML dataset (GSE21261) [22] (Figure 5B). To further characterize HDAC4 function in myeloid neoplasms, we analyzed HDAC4 expression levels in AML patients with normal karyotype versus healthy donors (GSE13159). Remarkably, HDAC4 expression levels in those patients were significantly lower than those in healthy individuals (Figure 5C). Importantly, lower than median levels of HDAC4 expression were associated with shorter overall patient survival (GSE12417 [23]) (Figure 5D). Taken together, these data support the idea that HDAC4 deficiency may promote disease progression in a subset of leukemia patients.

### A TET2 agonist in combination with HDACi potently inhibits MDS-L cell growth *in vivo*

**Figure 6:**
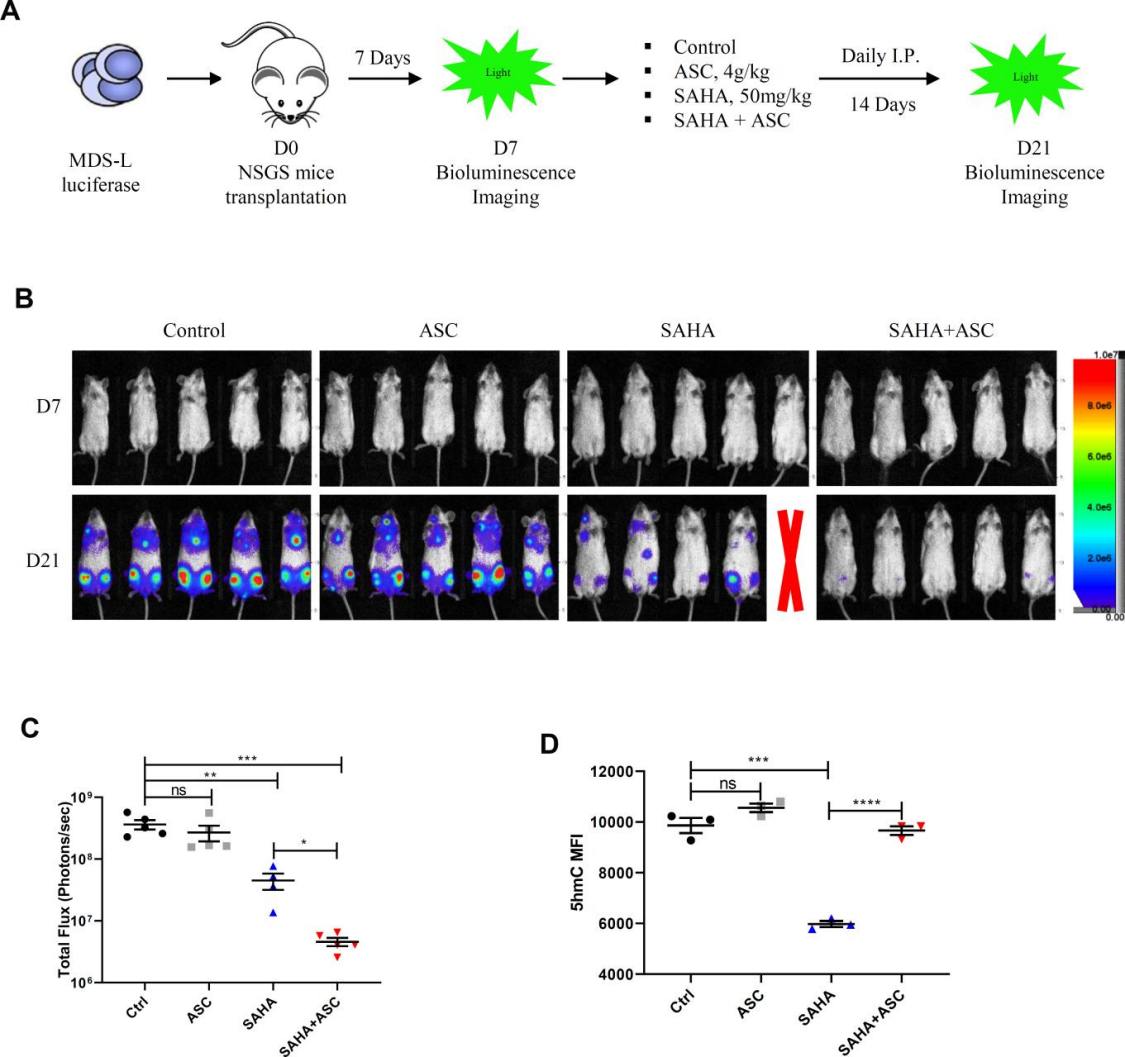
**TET2 agonist in combination with HDACi promotes potent anti-leukemia effects *in vivo*.** (**A**) Schematic showing experimental protocol. 2.5 x 10^6^ luciferase-expressing MDS-L cells were transplanted into NSGS recipient mice intravenously. Seven days later, baseline engraftment was detected by bioluminescence imaging. Drug treatment began on D7 via I.P. injection. MDS-L engraftment was detected at D21. (**B**) Pseudocolored bioluminescence images of NSGS mice transplanted with MDS-L cells and subjected to indicated drug treatment. Radiance is in units of “photons/second/cm2/steradian”. One mouse in the SAHA group died during drug administration (indicated by red X). (**C**) Total flux (photons/sec) of NSGS mice transplanted with MDS-L cells after indicated treatments. Signal intensity was quantified from each animal shown in panel b. (**D**) 5hmC levels (as quantified by flow cytometry) in peripheral blood cells of mice after indicated treatments.

Previous studies indicate that treatment with vitamin C, a TET2 agonist, enhances TET2 activity *in vivo* [24, 25], promoting DNA demethylation capacity and reversing aberrant self-renewal of leukemia initiating cells [25]. Thus, to assess effects of combining HDAC inhibition with vitamin C treatment, we transplanted luciferase-expressing MDS-L cells into NSGS mice to establish a MDS line xenograft model. Once we observed basal engraftment, mice were treated with vehicle, vitamin C (Ascorbate, ASC), SAHA or a combination of each (Figure 6A). Interestingly, combined SAHA and ASC treatment promoted greater inhibition in MDS-L engraftment than did treatment with either single drug (Figure 6B, 6C). We then assessed 5hmC levels in PB of treated mice and found that although total 5hmC levels decreased upon treatment with SAHA alone, this reduction was fully rescued after SAHA/ASC combination treatment (Figure 6D).

## DISCUSSION

Myeloid cancers including MDS and AML are highly heterogeneous diseases. For MDS, overall survival and risk of leukemic transformation for high-risk patients differ significantly from that of low-risk MDS [10]. Accumulating evidence indicates that TET2 deficiency leads to DNA hypermethylation, which is associated with poor prognosis of MDS and AML patients [26]. Herein, we report that HDACi treatment, primarily through HDAC4 inhibition, downregulates TET2 levels, thereby impairing active DNA demethylation capacity. Despite their effect as potent anti-cancer agents, such TET2 downregulation could be considered a drawback of pan-HDACi in this context. We found that lower HDAC4 expression levels correlated with shorter overall survival of a cohort of AML patients. Importantly, our critical finding is that in mouse models, vitamin C treatment prevented TET2 loss of activity induced by HDACi treatment and further enhanced anti-MDS efficacy of SAHA treatment. Overall, our results demonstrate a novel interplay between HDAC4 activity and TET2 function and provide a rationale for testing administration of a TET2 agonist in combination with HDACi in high-risk MDS and AML cases.

HDACs catalyze removal of acetyl groups from histones or non-histone proteins, resulting in chromatin condensation and transcriptional repression [13]. But their roles in cancers could be complicated. HDAC inhibitors like SAHA are known to serve as potent anti-cancer agents against various cancer subtypes [13]. SAHA is known to inhibit class I (mainly HDAC1/2/3) and class II HDACs, leading to hyperacetylation of many histones and non-histone proteins, and SAHA treatment could result in cell cycle arrest and cell death due to p21 upregulation and accumulation of reactive oxygen species (ROS) [27]. Moreover, in AML, HDAC1/2/3 can be aberrantly recruited by AML1-ETO fusion protein to repress AML1 target genes, resulting in cellular transformation [28, 29], indicating their oncogenic roles. In our study, we found that SAHA treatment alone modestly impaired MDS hematopoiesis of NHD13 mice, likely associated with HDAC1/2/3 inhibition. More importantly, we identified that HDAC4 positively regulates TET2 expression, and HDAC4 knockout significantly enhanced colony forming capacity of murine MDS cells, indicating that HDAC4 serves as a tumor-suppressor in the context of high-risk MDS or normal karyotype AML. Accordingly, we are currently expanding NHD13 crossed with HDAC4 KO mice in order to assess the direct impact of HDAC4 KO on MDS pathogenesis.

DNA methylation is a reversible epigenetic event regulating gene expression. As epigenetic erasers, TET proteins oxidize 5mCs and initiate the first step of active DNA demethylation [30]. TET2 is one of the most frequently mutated genes in myeloid malignancies, resulting in a dysregulated DNA methylation pattern in malignant cells. In addition to loss-of-function mutations, aberrant transcriptional repression and proteolytic activity also impair TET2 function in hematologic malignancies [31]. Previous studies report that IDAX and CXXC5 interact with TET2 and promote its degradation by caspase [32, 33]. Others have shown that TET2 is subject to calpain- and ubiquitination-mediated degradation [34, 35]. miR-22, a recognized oncogenic microRNA, is reportedly overexpressed in MDS and leukemia cells, negatively regulating TET2 mRNA levels [36]. Here, we show that HDAC4 is a novel regulator of TET2 at the transcriptional level and that HDAC4 deficiency decreases TET2 expression. Additionally, a functional interplay of HDAC4 and miR-22 has been reported [37]. However, whether HDAC4 regulates TET2 via miR-22 remains to be determined.

Utilization of high-doses of vitamin C as anti-cancer therapy has a long history [38]. Previous studies show that vitamin C may regulate multiple mechanisms, including redox imbalance, epigenetic reprogramming and oxygen-sensing [39]. A recent study revealed that vitamin C treatment mimicked the effect of TET2 restoration [25]. In our MDS-L xenografted NSGS model, combining vitamin C with SAHA restored 5hmC levels and reduced MDS-L engraftment relative to treatment with SAHA alone. We conclude that this synergy can be partially explained by vitamin C-mediated TET2 activity restoration, although further studies are required before other mechanisms can be excluded.

In conclusion, we observed that pan-HDACi treatment represses TET2 expression mainly through HDAC4 inhibition, leading to impaired DNA demethylation capacity in high-risk MDS/AML. Accordingly, vitamin C-induced TET2 restoration overcame inhibitory effects of HDACi on DNA demethylation and enhanced anti-leukemia efficacy *in vivo*. We conclude that restoring TET2 activity should be considered when using HDACi as a treatment option against high-risk MDS or AML.

## MATERIALS AND METHODS

### Mice

NUP98-HOXD13 (NHD13) mice and Vav1-Cre mice were purchased from Jackson Laboratory. HDAC4^fl/fl^ mice were purchased from Taconic Biosciences and bred at the City of Hope (COH) animal facility. All mice received autoclaved water and clean food, were subjected to 12-hour light/dark cycles, and kept in controlled ambient room temperature and air humidity conditions. All animal procedures were conducted in accordance with established institutional guidance and approved protocols of the Institutional Animal Care and Use Committee at COH. NHD13 mice were mated with HDAC4^fl/fl^/Vav1-Cre to obtain NHD13/HDAC4^wt/fl^/Vav1-Cre mice, and subsequently NHD13/HDAC4^fl/fl^/Vav1-Cre (NHD13/HDAC4 KO mice).

### *In vivo* treatment of transgenic NHD13 mice and MDS-L xenografted NSGS mice

Before drug treatment, NHD13 mice were divided into two groups with matched gender and age. Mice were then treated 4 weeks with SAHA (50mg/kg/day, I.P., n = 8) or vehicle (5% DMSO in 20% cyclodextrin, I.P., n = 8). Mice were then euthanized and PB and BM collected for further experiments. For NSGS mice, 2.5 x 10^6^ luciferase-expressing MDS-L cells were transplanted into recipients intravenously. Seven days later, basal engraftment was determined by bioluminescence imaging. Drug treatment began at day (D) 7 with vehicle (5% DMSO in 20% cyclodextrin, I.P., n = 5), ASC (4g/kg/day, I.P., n=5), SAHA (50mg/kg/day, I.P., n = 5) or SAHA + ASC (I.P., n=5). MDS-L engraftment was then evaluated at D21.

### *In vivo* bioluminescence imaging

NSGS mice were injected intraperitoneally with 150mg/kg D-luciferin (XenoLight). Ten minutes later, mice were anesthetized and imaged with Lago X (Spectral Instruments Imaging). Bioluminescent signals were quantified using Aura imaging software (Spectral Instruments Imaging). Total flux values were determined and are presented as photons (p)/second (sec).

### Flow cytometry

Mouse BM was aspirated from tibias and femurs, washed, and then filtered using 70-μm cell strainers. Cells were stained with required antibodies for 20 minutes at 4°C, washed and analyzed on a 5-laser, 15 detector LSRII. The DAPI (ThermoFisher Scientific)-negative population was selected as living cells. Mouse HSPCs were determined by staining with anti-mouse c-kit, Sca-1, CD16/32 and CD34 antibodies, biotin-linked lineage cocktails (including anti-mouse CD3, CD4, CD8, B220, IgM, CD19, CD11b, CD11c, NK1.1, Gr1, CD41 and ter119 antibodies (eBioscience)) and FITC-labeled streptavidin. The lineage^-^ c-kit^+^ Sca-1^-^ population was defined as LK, and the lineage^-^ c-kit^+^ Sca-1^+^ population as LSK. CMP, GMP and MEP populations were further gated from the LK population according to CD16/32 and CD34 expression. Mouse BM differentiation was evaluated using anti-mouse CD11b, Gr-1, B220, CD3 and Ter119 antibodies. Data were analyzed using BD FACS Diva or FlowJo software.

### BM c-kit^+^ cell selection

BM c-kit^+^ cells were isolated using mouse CD117 MicroBeads (Miltenyi Biotec) according to the kit protocol. Briefly, total BM cells were counted and resuspended in MACS buffer. After staining with CD117 microbeads (20μL per 10^7^ total cells, 15 min at 4°C), cells were washed and applied onto MS columns, which were rinsed and placed in a magnetic field. Columns were washed three times with 500 μL buffer and then removed from the separator. Then magnetically-labeled cells were flushed out with 1mL buffer. c-kit^+^ cells were counted and used for further experiments.

### Intracellular 5hmC staining

For intracellular staining, mouse BM was first stained with HSC surface markers, as described above. Peripheral blood from NSGS mice was lysed with ammonium chloride potassium (ACK) buffer, and remaining MNC cells were washed and fixed with 4% paraformaldehyde in the dark for 15 minutes and permeabilized with 0.5% Saponin permeabilization solution (BD Bioscience) for 1 hour at room temperature. After washing, cells were labeled with rabbit anti-5hmC antibody (active motif, 1/200, 30 mins at 4 °C) or isotype control and then incubated in Alexa 488-conjugated goat anti-rabbit antibody (Invitrogen, 1/1000, 30 mins at 4 °C), washed and analyzed by flow cytometry.

### 5hmC quantification by dot blot and ELISA

5-hmC levels in treated cells were detected by dot blots and ELISA as described [8]. For dot blots, DNA samples were isolated using a DNeasy Blood and Tissue kit (Qiagen). Denatured DNA and two-fold serial dilutions were spotted onto nitrocellulose membranes, and after UV cross-linking membranes were blocked and incubated with anti-5hmC antibody (1/1000) overnight at 4°C. Next, membranes were washed and incubated with HRP-conjugated second antibody for 1 hour at room temperature, and signal density was detected using an ECL kit (ThermoFisher Scientific). To ensure equal loading of total DNA, the same membranes were stained in 0.02% methylene blue in 0.3M sodium acetate. To quantify 5hmC, a MethylFlash Global DNA Hydroxymethylation ELISA Easy Kit (Epigentek) was used according to the suggested protocol. Briefly, equal amounts (100ng) of DNA were added, and the initial incubation time was 90 min and final developing time 10 min. Absorbance at 450 nm was determined.

### Western blotting

Western blotting was performed as described [8]. Cells were lysed in CytoBuster Protein Extraction Reagent (Millipore), and boiled lysates were then resolved on 7.5% or 12% SDS-PAGE gels and transferred to nitrocellulose membranes (Bio-Rad). The following primary antibodies were used: rabbit anti-mouse Tet2 (ab124297, Abcam), rabbit anti-human Tet2 (GTX124205, GeneTex), rabbit anti-human/mouse HDAC4 (15164, Cell Signaling Technology), rabbit anti-human/mouse H_3_K_27_ac (8173S, Cell Signaling Technology), and mouse monoclonal anti-beta-actin (sc-69879, Santa Cruz Biotechnology).

### RT-qPCR analysis

Total RNA was isolated from cells using Trizol reagent (Invitrogen) according to the manufacturer's protocol. cDNA was amplified using a High Capacity cDNA Reverse Transcription Kit (Applied Biosystems). Quantitative real time PCR was performed using TaqMan Fast Advanced Master Mix (Applied Biosystems) with 0.2μM Taqman probe (Life Technologies). PCR amplification was carried out in a QuantStudio 7 Flex Real-Time PCR system (Life Biotechnology). Relative transcript levels were normalized to GAPDH levels.

### hMeDIP-qPCR analysis

hMeDIP assays were performed based on the manufacturer’s instructions using the SimpleDIP kit, as described [8]. DNA was denatured and IP’d with anti-5hmC or IgG control antibodies and protein G magnetic beads. After three washes, 5hmC-modified DNA was eluted from beads, purified and used as the template in qPCR.

### Cell culture

The human MDS cell line MDS-L was developed and provided by Dr. Kaoru Tohyama [18]. MDS-L cells were cultured in RPMI 1640 with 10% FBS and 1% penicillin-streptomycin in the presence of 30 ng/mL human recombinant human IL-3. Nomo-1 and other AML cell lines were cultured in 10% FBS/RPMI 1640 medium.

### Lentivirus transduction and cell transfection

First, 293T cells were transiently transfected with pCDH plasmids and pMD2G/pSAPX2 packaging plasmids, and then 24-36 hours later, supernatants containing replication-incompetent lentiviruses were collected and concentrated using PEG-it (System Biosciences). After titration, target cells were exposed to virus (MOI=10), and 48 hours later, cells were sorted by flow cytometry based on GFP/RFP expression. For siRNA transfection, Nomo-1 cells were transfected with HDAC1-8 siRNA (Dharmacon) by nucleofection (Lonza). A FAM-conjugated siRNA control was included, and FAM positivity above 90% was considered successful transfection.

### *In vitro* colony forming assays

NHD13/HDAC4 WT or KO BM were obtained and suspended in methylcellulose medium (M3434, StemCell Technologies) and seeded into plates (5 × 10^4^ per well) according to manufacturer’s protocol. Colonies were counted 7 days later. For replating assays, cells from each plate were harvested and replated at 5 × 10^4^ per well.

### Cell viability analysis

Cell viability was determined using the Cell Titer-Glo Luminescent Cell Viability Assay Kit (G7570, Promega). Briefly, cells were cultured under different treatment conditions and transferred to 96-well plates for 30 min. Kit substrate and buffer reagents were then added to each well and mixed to allow cell lysis. Plates were read on a Microplate reader (Beckman culture DTX880). Results were expressed as a percentage of untreated cells, for three replicates.

### Clinical outcome analysis

Clinical outcome analysis was performed as described [8]. Briefly, RNA sequencing data form normal karyotype AML bone marrow mononuclear cells (GSE12417, 163 patients with survival information) was obtained. Patients are separated into 3 groups based on the K-means clustering approach (Hartigan-Wong algorithm). Then, the survival curves are created using Function “survfit” (in R package “survival”) from Kaplan-Meier formula. Finally, the log-rank p-value is calculated by Function “survdiff” (in R package “survival”) to test the differences among 3 survival curves using Gp family of tests.

### Statistical analysis

Data obtained from independent experiments were expressed as means ± SEM, and *n* represented the number of samples. Two-tailed Student’s *t*-tests were used to compare differences between groups. GraphPad Prism 8 software (La Jolla, CA) was used to assess statistical analyses. *P* < 0.05 represented a statistically significant difference.

### Ethics statement

All animal procedures were conducted in accordance with established institutional guidance and approved protocols of the Institutional Animal Care and Use Committee at COH.

## Supplementary Material

Supplementary Figures
