# Supplementary material for: HDAC4 inhibition disrupts TET2 function in high-risk MDS and AML
Source: Aging (Albany NY). 2020 Jul 29;12(17):16759–74. doi: 10.18632/aging.103605 (PMC7521497; doi:10.18632/aging.103605)
Supplement: Supplementary Figures [file aging-12-103605-s001..pdf]

SUPPLEMENTARY FIGURES

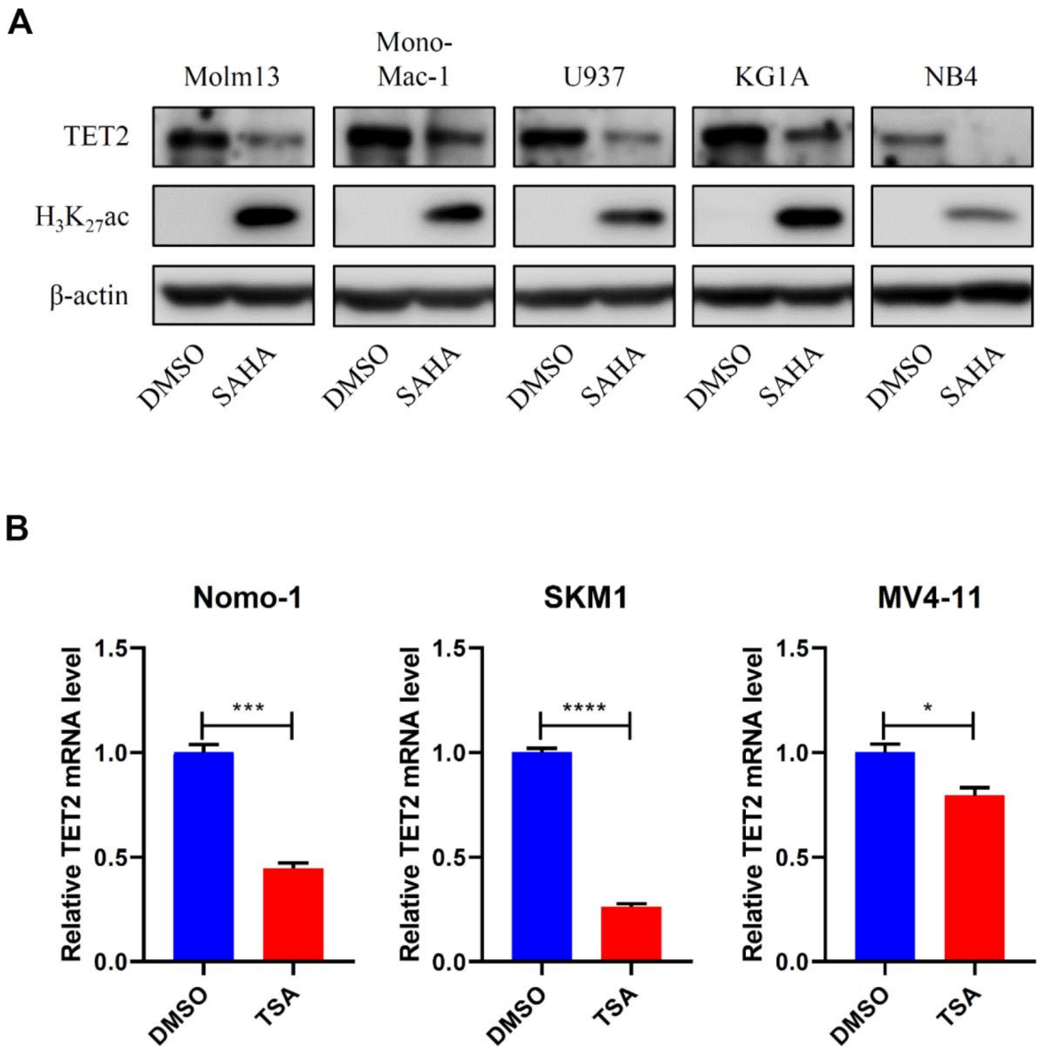

Supplementary Figure 1. HDACi treatment promotes DNA hypermethylation. (**A**) Indicated AML lines were treated with 2 μM SAHA for 24 hours, and TET2 protein levels were detected via Western blot. H3K27Ac served as a positive control. (**B**) Indicated AML lines were treated with 1 μM TSA for 24 hours, and TET2 mRNA levels were determined by RT-qPCR.

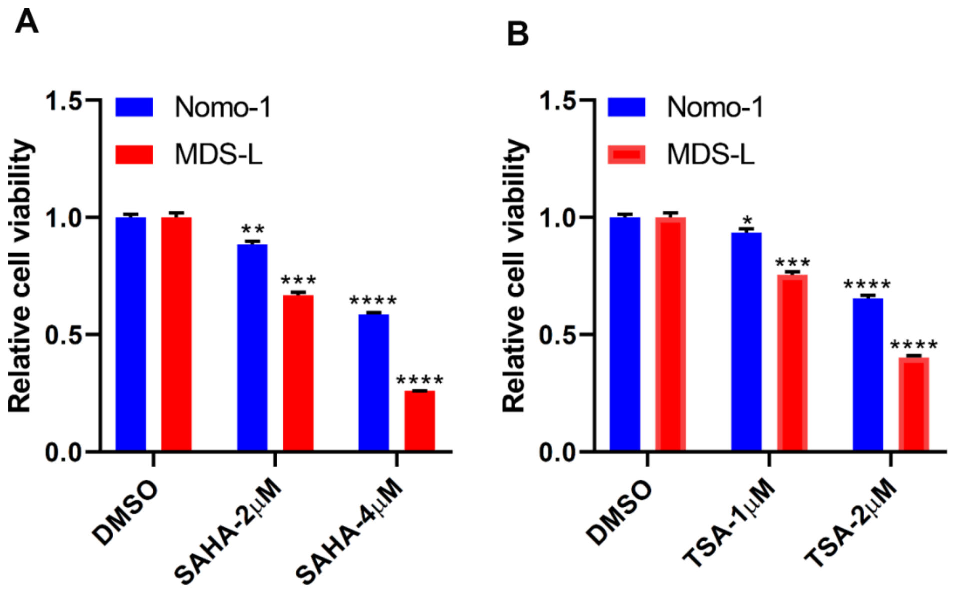

Supplementary Figure 2. HDACi treatment reduces MDS/AML cell viability. (**A**) MDS-L and Nomo-1 cells were treated with SAHA for 24 hours, and cell viability was determined using a Cell Titer-Glo kit. (**B**) MDS-L and Nomo-1 cells were treated with TSA for 24 hours, and cell viability was determined as in (**A**).
